# Corporate Social Responsibility: What Are Foodservice Companies Reporting?

**DOI:** 10.3390/ijerph19159214

**Published:** 2022-07-28

**Authors:** Minseong Kim, Ho-Seok Kim

**Affiliations:** 1 Department of Management & Marketing, College of Business, Louisiana State University Shreveport, Shreveport, LA 71115, USA; 2 Department of Hotel Culinary Art and Hospitality Management, Catholic Kwandong University, 24, Beomil-ro 579 beon-gil, Gangneung-si 25601, Korea; hskim@cku.ac.kr

**Keywords:** corporate social responsibility, CSR, foodservice, hospitality and tourism, sustainability index, environment, societal marketing

## Abstract

This study identified aspects of corporate social responsibility (CSR) activities using online communication tools (i.e., websites and online CSR reports) with an emphasis on the foodservice industry and compared quick-service restaurants and dessert cafes. With the content analysis of 48 foodservice companies, the community, environment, marketplace, vision and values, food, and workforce-centered CSR activities implemented by the selected foodservice companies were measured. In addition, the types CSR information delivered to customers employed by the foodservice companies were investigated. Lastly, there were significant differences between two segments in the foodservice industry in some aspects of CSR activities and types of CSR activities.

## 1. Introduction

Since the 1990s, corporate social responsibility (CSR) has attracted media attention in the United States, and it has played a role in the public relations campaign arsenal of a plethora of corporations [1]. A common assumption in an ideal world is that firms need to initiate CSR activities for their business as a consequence of an ethical obligation, which is both externally and internally encouraged. Scholars and practitioners have supported this notion by suggesting that there are a variety of reasons for acting in a socially responsible way. For instance, CSR initiatives can increase employee organizational commitment [2], economic profits [3], and public scrutiny [1] as well as improve the welfare of society and investor relations [4], which includes the perspective that CSR activities are right things to do. However, prior research in this field focused primarily on the consequences of CSR initiatives of corporations from the perspectives of either internal consumers (i.e., employees) or external consumers (i.e., customers) [1,2,3,4,5]. In other words, although previous empirical studies emphasized how internal and external consumers perceive CSR activities, they neglected how corporations deliver and/or report their CSR initiatives to stakeholders via various communication channels. Hence, this study proposes that scholars and practitioners need to understand how the CSR-related information is currently delivered and reported by corporations to various stakeholders before conducting empirical studies. The approach of this study helps scholars and practitioners to clearly prepare for measuring and investigating various stakeholders’ perceptions of CSR initiatives for their future research, leading to reduced potential errors and risks (e.g., conceptual and empirical gaps between CSR initiatives of corporations and stakeholders’ perceptions of CSR initiatives) [6].

In particular, foodservice enterprises have sometimes faced CSR dilemmas, even frequently, because of circumstances which are not entirely under their control. For instance, most customers believed that fries from McDonald’s were vegetarian friendly after this corporation informed their customers of their decision to cook fries in vegetarian oil. Unfortunately, McDonald’s actually used beef tallow for its fries and did not disclose this information to customers. McDonalds’ later apologized for their socially irresponsible actions, and they should pay religious and vegetarian groups USD 10 million for misleading customers regarding its fries’ content [5]. This scandal depicts the harmful results of irresponsible actions in the foodservice industry. The continued growth of the foodservice industry has resulted in a dramatic increase in various stakeholders’ concerns regarding its negative influences on the natural environment and society including the generation of food waste/disposable products and overuse of natural resources [7]. This ongoing trend motivates foodservice companies to respond to stakeholder demands through efforts to spread societal and environmental benefits [7]. Given the consequences, many foodservice enterprises have already tried to institutionalize CSR initiatives by publishing their CSR reports, demonstrating their CSR activities on official websites, or by creating independent websites emphasizing their CSR activities in several categories (e.g., food-related, community-related, or ingredient-related CSR activities) [5].

The online information regarding CSR activities by foodservice companies will enhance their business performance and can better respond to external demands and pressure from various stakeholders (e.g., government, community, supplier, and consumers) [7]. The online reports and information on foodservice companies’ websites target stakeholders and have been used as communication tools to indicate their CSR activities in an easily accessible manner for both existing and potential customers [8,9]. Given the growing significance of CSR and online information in the foodservice industry, an investigation of CSR activities implemented by foodservice companies needs to be performed to understand in-depth their self-presentation of CSR initiatives. 

CSR activities have been expressed from a variety of aspects including natural environment-centered, community-centered, and employee-centered CSR activities. For example, some foodservice companies engaging in CSR activities tend to focus more on community-centered aspects of CSR activities than on environment-centered ones (e.g., Sonic Drive-In Restaurant). Other foodservice enterprises implementing CSR activities tend to emphasize various aspects of CSR activities (e.g., environmental, social, and governance aspects by McDonald’s). This inconsistent information regarding foodservice companies’ CSR activities may become a barrier to understanding CSR activities among customers which, in turn, may have a harmful effect on the foodservice companies’ business performance in the long term. However, there is still a lack of empirical research with regards to aspects of foodservice enterprises’ CSR activities in the public domain. Thus, the purposes of the current research were to (1) examine how foodservice companies communicate CSR activities with stakeholders; (2) identify aspects of foodservice operators’ CSR initiatives as exemplified via a systemic review of their official websites or CSR reports employing a content analysis approach; (3) determine what types of information foodservice companies provide; (4) investigate how identified aspects of foodservice companies’ CSR activities differ in different segments. In order to achieve the research purposes, this study emphasized the CSR activities of the top foodservice franchise companies listed in *Entrepreneur*. This study is outlined as follows: Section 2 contains a literature review focusing on the definition of CSR within a hospitality context; Section 3 presents the methodology (i.e., data collection and codebook); Section 4 discusses the findings including the data analysis and empirical results; Section 5 is the discussion and implications; Section 6 provides the conclusion and limitations.

## 2. Literature Review

### 2.1. Corporate Social Responsibility (CSR) Defined

CSR has been called a variety of terminologies such as corporate sustainability as well as corporate citizenship and social responsibility. The general definition of CSR was proposed by the Dow Jones Sustainability Index (DJSI) as follows:

“CSR is a business approach that creates long-term shareholder value by embracing opportunities and managing risks deriving from economic, environmental and social developments. CSR leaders achieve long-term shareholder value by gearing their strategies and management to harness the market’s potential for sustainability products and services while at the same time successfully reducing and avoiding sustainability costs and risks.”

In addition to the DJSI, the business in the community (BITC) defines CSR as “the management of a company’s positive impact on society and the environment through its operations, products or services and through its interaction with key stakeholders such as employees, customers, investors and suppliers”. The concept of CSR has been formulated by several scholars such as Carroll [10]. His study established businesses initiating CSR activities attending to “economic, legal, ethical, and discretionary (philanthropic) expectations that society has of organizations at a given point in time” (p. 499). Economic responsibility is associated with beliefs that companies need to produce competitive and profitable services and/or products in order to meet the needs of both customers and society. Legal responsibility is based on companies’ obligation of obeying regulations and rules and to operate their business within legal boundaries. Ethical responsibility goes beyond legal regulations or obligations, reflecting implicit social values and norms. Finally, philanthropic responsibility is companies’ activities contributing to goodwill and human welfare by giving back to society financial and nonfinancial resources [10]. This notion is based on a corporation’s responsibility for society and duty of doing what society expects. Donaldson and Preston [11] and Clarkson [12], on the other hand, believe that a corporation does not have responsibility to society but possesses responsibility for its stakeholders, which include suppliers, employees, communities, and customers, by viewing CSR initiatives from this standpoint. However, all previous studies have agreed that CSR’s definition is based on the notion that a firm needs to engage in CSR initiatives as part of its business strategy. The consequence of initiating CSR activities as business strategies is that they provide the host corporations with economic and social benefits in competitive market conditions [13]. Based on the above definitions of CSR, this study operationalized foodservice companies’ CSR initiatives as a business strategy that serves local communities, commits to employee relations and consumer rights, and reduces environmental issues and health concerns, leading stakeholders to have better perceptions of the CSR initiatives.

### 2.2. The Scope of CSR Reporting

From the general public’s perspective, there have been structures on how to gauge the absence and/or presence of a corporation’s CSR activities in terms of environmental and social practices. In the case of the United States, generally accepted accounting principles (GAAP) indicate several policies and regulations in terms of the disclosure of a corporation’s financial information with an emphasis on the public domain. Although an annual report has been traditionally employed for the disclosure of a corporation’s financial information, providing disclosure of environmental and social compliance in an annual report is not uncommon for a corporation. Embracing social and environmental aspects tends not to be an anomaly due to the fact that they are considered indicators of a corporation’s overall strengths in the market [14]. Hence, many corporations have shared how well their CSR initiatives are aligned with the 17 Sustainable Development Goals of the United Nations and indicate how each goal is implemented and measured on their CSR or ESG (environment, social, and governance) report and/or official websites and social media pages. Each indicator for a corporations’ financial and nonfinancial performances consists of both qualitative and quantitative methods, which are clearly delivered by their various stakeholders based on each section (e.g., people, planet, and profit). In addition, some companies have partnerships with other companies by working toward objectively measuring and reporting financial performances as well as social performances to the public, which are generated primarily by CSR initiatives. In the case of the European Union, the European Modernization Directive has been activated, which requires all countries to draw up a bill for the explicit objectives of reporting environmental and employee matters (Directive 2014/95/EU (Non-Financial Reporting Directive—NFRD) and recent regulatory developments, the international initiative TCFD, the CSRD package with the addition of ESRS, the Taxonomy system, or SFDR). For example, France, the Netherlands, and the United Kingdom are complying with the Directive. More specifically, the United Kingdom passed the operating and financial review (OFR) bill requiring corporations to release a report on their environmental and social activities. The OFR has been expected to be a benchmark that other member countries will employ as a standard [15].

It has appeared that emphasizing CSR has ramped up to become a movement globally. Scholars and practitioners argue that CSR initiatives serve as a solution to address ongoing and forthcoming social and environmental issues [16]. According to the state of global social and environmental reports, for example, approximately 65 percent of the top 100 companies around the world are already engaged in releasing CSR reports through some form on the Internet [14]. Additionally, Esrock and Leichty [17] indicated that approximately 90 percent of corporations’ official websites address their CSR initiatives with an emphasis on environmental concern, public educational support, and community involvement. However, it appears that the interest in releasing a CSR report on a website is led by the main objective of establishing and enhancing a positive public image more than anything else. Nevertheless, the interest tends to be complementary to the publication of a CSR report on the website [17].

### 2.3. The State of CSR in the Hospitality and Tourism Industry

The hospitality and tourism industry fields have emphasized the efficient use of energy and technology as well as environmental issues since 1992 [18]. This focus was enhanced on an international scale via implementing Agenda 21. This program was established by the World Tourism Organization (WTO), the World Travel and Tourism Council (WTTC), and the Earth Council and based on international guidelines associated with sustainable business. Then, the WTO established the Global Code of Ethics for Tourism (GCET), a “comprehensive set of ten principles whose purpose is to guide stakeholders in tourism development” [19]. Even though the ten principles are not legal requirements, they play a role as guidelines to tourism operators, local communities, central governments, and tourists with respect to preservation and protections of the natural environment. 

In the case of Europe, two hospitality institutions established a CSR initiative for the hospitality industry. The initiative drafts compliance indicators of working conditions, nondiscrimination, equal opportunity, fair pay, health and safety, lifelong learning and vocational training, and relationships between employees and employers that embrace all levels [1]. The initiative has been significant to note for corporations in the European arena, since it reflects the industry’s engagement in CSR activities in contrast with governmental compliance directives that are externally derived. The literature in the hospitality and tourism fields has also concluded that CSR has received considerable attention from hospitality and tourism enterprises and institutions through public media outlets over the past decade. 

Within the foodservice industry, which is the context of this study, it has been suggested that the disclosure of companies’ CSR activities can ultimately motivate their employees to become committed to their tasks as well as attract potential customers [19,20]. In addition, Lee, Singal, and Kang [21] found that foodservice companies’ CSR activities can create competitive advantages and corporate values, since investment in CSR enhances corporate governance, employment, safety, and product quality. Especially, the CSR activities can enhance the level of customer satisfaction with foodservice companies’ products and services as well [22]. The information about foodservice companies’ CSR activities can effectively create long-term relationships with stakeholders (e.g., company identification, good reputation, and positive image) [6,23,24,25]. This is primarily because the foodservice companies are dependent on physical and natural resources as well as human resources by generating relatively greater (both positive and negative) influences on the natural environment and society than companies in other sectors, resulting in a higher level of public criticism [6]. The CSR programs are significantly associated with the broader CSR initiatives, which occur all around the world and become more codified within the foodservice industry. However, there are too many types of online communication tools to deliver information regarding a foodservice companies’ CSR activities to their stakeholders. In addition, foodservice companies have reported different aspects of CSR activities in the most favorable manner. In particular, Talwar et al. [26] highlighted that foodservice companies should focus on the role of consumers’ health consciousness in evaluating CSR effectiveness, leading consumers to seek knowledge and information about the companies’ products. 

This study was based on stakeholder theory that emphasizes the roles of suppliers, employees, shareholders, and customers who make direct and/or indirect managerial decisions for a company [27,28]. More interestingly, the stakeholder theory also focuses on the aspects of society and the environment as “silent stakeholders” that exist inside and outside of a company, responsibly managing the company [27]. Therefore, stakeholder theory has been incorporated into CSR studies as a new way to broadly think about a company’s social and environmental responsibilities beyond its responsibilities for external and internal customers as well as suppliers [28]. This is because the fundamental notion of stakeholder theory is that one stakeholder’s satisfaction/needs can gradually turn their attention to other stakeholders’ considerations for mutual success in the natural environment and society [27,28]. Based on the aforementioned notions and real situation, this study formulated the following research questions:

**RQ1.**  *How do top foodservice companies communicate CSR activities with customers (e.g., CSR report, another website focusing on CSR activities, and CSR categories on their official website)?*

**RQ2.**  *What aspects of CSR information do they provide (e.g., food, people, community, and environment)? Are foodservice companies focusing more on internal and external customers than on community and environment?*

**RQ3.**  *What types of information do they provide?*

**RQ4.**  *How do aspects of CSR information differ in different segments (e.g., quick-service, casual, and fine dining)?*

## 3. Methodology

### 3.1. Sampling and Measurement

Content analysis was employed to identify and depict patterns on the identified foodservice companies’ official websites or CSR reports (depending on what they have published). Neuman [29] stated that “content analysis is a technique for gathering and analyzing the content of text. The content refers to words, meanings, pictures, symbols, ideas, themes, or nay message that can be communicated” (p. 219). This study identified themes through the content analysis procedure by applying them to data analyzed. CSR consists of various dimensions from the natural environment to employees’ responsible treatment meaning that each corporation tends to differently label similar CSR activities depending on its sources. Regarding employees’ socially responsible treatment, for instance, it has been categorized as employees [12], employee well-being [30], human resources [31], and workforce [1]. To identify aspects for the foodservice industry, this study analyzed prior studies as well as official websites or CSR reports of selected foodservice companies. This study identified categories which well fit CSR activities foodservice companies are reporting on the websites. The categories would be “community”, “environment”, “marketplace”, “vision and values”, and “workforce” [1]. The categories come from prior studies within the hospitality industry. To explain the categories’ content, types of aspects addressed in Table 1.

The research sample consisted of the top foodservice companies as listed in *Entrepreneur* Franchise 500 in 2021 (https://www.entrepreneur.com/franchise500, accessed on 1 July 2021) with a purposive sampling approach. This website compares franchise firms on specified factors (i.e., “costs and fees”, “size and growth”, “support”, “brand strength”, and “financial strength and stability”). Based on the sampling approach, this study only identified the top 48 foodservice companies among the top 500 franchise companies in 2021. The research sample is shown in Table 2.

This study conducted a content analysis of the selected foodservice companies’ official websites with Statistical Package for the Social Sciences (SPSS) 22.0 to measure and analyze their CSR categories. This study downloaded the selected foodservice companies’ annual CSR reports and accessed CSR-related information on their official websites to investigate each content for data analysis (e.g., [1] if a company uses an official website for its CSR communication, this study investigated the official website; [2] if a company publishes an annual CSR report, this study examined the CSR report; [3] if a company uses an official website and publishes an annual report for its CSR communication, this study investigated CSR-related content from its official website and annual report simultaneously). After identifying each company’s CSR activities on their website and/or annual report, this study sorted the information into common themes based on the codebook.

### 3.2. Intercoder Reliability

The selected foodservice companies were randomly divided and assigned to two independent coders: one of the authors and an independent coder from the University of Kentucky. The first coder, the author, investigated 28 foodservice companies out of the selected foodservice companies, and the second coder, the independent coder from the University of Kentucky, examined 30 foodservice companies out of the selected foodservice companies based on the codebook created by the authors. The two coders’ coding of 10 overlapped foodservice companies (*n* = 10) were compared to check intercoder reliability before proceeding with data analyses to answer research questions. Specifically, 10 overlapped foodservice companies were cross-referenced for intercoder reliability to rigorously check whether the coding procedure between two independent coders was appropriate for data analyses. Cohen’s Kappa was employed to calculate reliabilities of nominal data [32] which enables researchers to check the number of chance agreements between two coders [33]. The appropriate approach is not to report one reliability for all the variables, but to report reliability for each variable (i.e., each CSR aspect in this study) [34]. The results of Cohen’s Kappa showed that the values of all variables (i.e., community = 0.812, *p* < 0.001, environment = 0.800, *p* < 0.001, marketplace = 0.792, *p* < 0.001, vision and values = 0.827, *p* < 0.001, workforce = 0.780, *p* < 0.001, food = 0.717, *p* < 0.001, and type of information: community = 0.871, *p* < 0.001, environment = 0.861, *p* < 0.001, marketplace = 0.837, *p* < 0.001, vision and values = 0.770, *p* < 0.001, workforce = 0.879, *p* < 0.001, and food = 0.916, *p* < 0.001), satisfying an acceptable score that ranged from 0.70 to 1 (see Table 3) [32]. In the case of checking reliabilities of nominal data, the percentage agreement between two coders is considered a good index [34]. After checking reliabilities, this study performed data analyses with SPSS 22.0 to answer research questions 1, 2, 3, and 4.

## 4. Findings

### 4.1. Communication Tool for CSR Activities

Research Question 1 asked how top foodservice companies communicated CSR activities with customers. As shown in Table 4, of the communication tools for foodservice companies’ CSR activities, official websites with CSR categories accounted for 62.5% (*n* = 30), followed by CSR reports 14.6% (*n* = 7) and independent CSR websites 12.5% (*n* = 6). Among the identified foodservice companies, 31.3% did not use any type of communication tools to deliver information about CSR activities to customers. Compared to CSR reports and independent CSR websites, official websites with CSR categories were mostly used by foodservice companies (official website with CSR categories 62.5% vs. CSR report 14.6%, independent CSR website 12.5%).

### 4.2. Aspects of CSR Information

Research Question 2 examined the aspects of CSR information that they did provide. This research question was related to foodservice companies’ foci on internal and external customers rather than community and environment, because foodservice companies should care more about food-related health and safety for external customers and the well-being of internal customers (i.e., reliance on human resources for service delivery) than other companies in different industries [6]. As exhibited in Table 5, of the community-centered CSR activities, charitable donations (69.7%, *n* = 23), corporate giving (78.8%, *n* = 26), education (54.5%, *n* = 18), and community welfare (78.8%, *n* = 26) dominated the foodservice companies’ activities in comparison with donations in kind (3.0%, *n* = 1), grants (15.2%, *n* = 5), national welfare (12.1%, *n* = 4), world welfare (3.0%, *n* = 1), and local regeneration (6.1%, *n* = 2). Interestingly, compared to other volunteerism types (i.e., national-level volunteerism and world-level volunteerism), some of the foodservice companies implemented local volunteerism (39.4%, *n* = 13).

As shown in Table 6, among the natural environment-focused CSR activities, approximately 25% of foodservice companies implemented energy management (24.2%, *n* = 8), recycling (27.3%, *n* = 9), water conservation (24.2%, *n* = 8), pollution control (24.2%, *n* = 8), and waste management (18.2%, *n* = 6).

As demonstrated in Table 7, among the marketplace-centered CSR activities, providing a product of value (72.7%, *n* = 24) dominated foodservice company activities compared to relationships with shareholders (6.1%, *n* = 2) and ethical advertising (0.0%, *n* = 0). In addition, approximately 30% of foodservice companies focused on relationships with guests (33.3%, *n* = 11) and relationships with supplies (27.3%, *n* = 9).

As depicted in Table 8, of the vision and values-focused CSR activities, building brand trust was the dominant aspect among foodservice companies (51.5%, *n* = 17) in comparison to ethical behavior (9.1%, *n* = 3) and fairness (12.1%, *n* = 4). Approximately 30% to 35% of foodservice companies established a code of conduct (27.3%, *n* = 9), enduring values (24.2%, *n* = 8), and self-regulation (36.4%, *n* = 12).

As shown in Table 9, among the food-related CSR activities, approximately 60% of foodservice companies emphasized sourcing (48.5%, *n* = 16), ingredient and materials (60.6%, *n* = 20), and food quality and choice (57.6%, *n* = 19). Compared to other CSR activities, food safety accounted for approximately 39.4% (*n* = 13).

As exhibited in Table 10, of the workforce-centered CSR activities, approximately 35% to 40% of foodservice companies focused on career planning (33.3%, *n* = 11), training (42.4%, *n* = 14), employee assistance program (30.3%, *n* = 10), compensation and rewards (39.4%, *n* = 13), fair and equitable benefits (30.3%, *n* = 10), diversity and equal opportunity (36.4%, *n* = 12), and employee communication (33.3%, *n* = 11). However, foodservice companies did not do much related to daycare and family accommodations for their employees compared to other aspects (9.1%, *n* = 3).

### 4.3. Types of Information for Delivering CSR Activities

Research Question 3 investigated the types of information foodservice companies provided. As exhibited in Table 11, among the types of information regarding community-based CSR activities, pictures were the dominant type of information for delivering CSR activities (63.5%, *n* = 21) compared to others (i.e., figures, tables, statistical data, citations, and text only). Regarding types of information regarding environment-centered CSR activities, statistical data (27.3%, *n* = 9) and pictures (21.2%, *n* = 7) dominated foodservice companies’ methods of delivering CSR activities to customers compared to other types. In terms of the marketplace-focused CSR activities, pictures (21.2%, *n* = 7) and words (27.4%, *n* = 9) were used more by foodservice companies than others (i.e., figures, tables, statistical data, and citations). With respect to the vision and values-related CSR activities, text only was primarily used by foodservice companies than others (33.3%, *n* = 11). With regard to the food-focused CSR activities, statistical data (30.3%, *n* = 10), pictures (30.3%, *n* = 10), and words (27.3%, *n* = 9) were the dominant types of information that delivered CSR activities to customers. Lastly, regarding the workforce-focused CSR activities, citations (18.2%, *n* = 6), pictures (24.2%, *n* = 8), and words (33.3%, *n* = 11) were more employed by foodservice companies than other types.

### 4.4. Different Segments in the Foodservice Industry

Research Question 4 was related to differences between segments in the foodservice industry (i.e., quick-service restaurant vs. dessert cafe). Chi-square analysis was conducted to investigate significant differences between quick-service restaurants and dessert cafes (see Table 12). There were significant differences between two different segments in the foodservice industry: corporate giving χ^2^(1) = 3.636, *p* < 0.10; community welfare χ^2^(1) = 3.636, *p* < 0.10; fairness χ^2^(1) = 3.098, *p* < 0.10; community—statistical data χ^2^(1) = 4.591, *p* < 0.05; community—picture χ^2^(1) = 6.991, *p* < 0.01; environment—table χ^2^(1) = 3.098, *p* < 0.10; food—table χ^2^(1) = 3.098, *p* < 0.10. More specifically, corporate giving, community welfare, and fairness were implemented more by quick-service restaurants than by dessert cafes. Regarding the types of information, statistical data for community, tables for natural environment, and tables for food were employed more by dessert cafes than by quick-service restaurants, while pictures for community were used more by quick-service restaurants than by dessert cafes.

## 5. Discussion

This study provides theoretical contributions to the existing CSR literature in the foodservice context. First, this study identified and measured aspects of CSR activities (i.e., community, environment, marketplace, vision and values, workforce, and food) within the foodservice industry. This approach is aligned with prior empirical CSR research within the foodservice context, broadly conceptualizing CSR initiatives as social, environmental, and governance [6]. In other words, CSR activities of foodservice companies did not emphasize one aspect but a wide range of aspects from internal levels to external levels of the companies, and the reporting contributions of each aspect of CSR activities on their websites and/or reports is particularly important. Although Carroll’s CSR model consisting of four dimensions (i.e., economic, legal, ethical, and philanthropic responsibilities) is outdated and was developed based on the manufacturing industry, several studies have still employed the model for the foodservice industry [5,35]. For example, Kim et al. [35] found that strategic environmental marketing was significantly influenced by economic, legal, ethical, and philanthropic CSR dimensions, while tactical environmental marketing was significantly affected by economic, ethical, and philanthropic CSR aspects. Hence, prior research has also argued that more studies on the additional aspects of CSR activities with an emphasis on the foodservice context need to be conducted. From a theoretical perspective, this study expands stakeholder theory by considering the societal and environmental aspects as new stakeholders in the hospitality industry [28,35]. Therefore, this study provides several new aspects of foodservice companies’ CSR activities via content analysis based on the Carroll’s model and the hospitality research [1] as well as stakeholder theory [25].

Second, based on the segment of the foodservice industry (i.e., quick-service, casual, fine dining, and dessert café), this study found significant differences between two segments (i.e., quick-service restaurant vs. dessert cafe) in aspects and information of foodservice companies’ CSR activities. The empirical research of Kim and Stepchenkova [36] also examined the significant differences in perceptions between quick-service consumers and casual dining consumers in the social media context. The CSR activities within the foodservice context have been investigated to predict companies’ business performance and/or consumers’ favorable perception, attitude, and behavior toward the companies [37]. To date, researchers have emphasized the important role of CSR activities in enhancing a brand reputation and increasing a positive brand image with an emphasis on either fine-dining restaurants or quick-service restaurants. However, little research has focused on the investigation of differences between segments within the foodservice industry. Each restaurant sector in the foodservice industry provides consumers with different products and experiences, leading to different types of CSR activities and CSR values [36]. From another theoretical standpoint, if scholars overlook the different characteristics of each restaurant sector (e.g., fine dining vs. casual dining vs. quick-service vs. dessert cafe), empirical findings of their research may result in a misunderstanding of the CSR initiatives within the restaurant context. Responding to this research gap, this study highlighted the significant differences between quick-service restaurants and dessert cafés in terms of aspects and information about foodservice companies’ CSR activities. Therefore, it may be essential that different segments of foodservice companies implement and deliver different aspects and information about CSR activities depending on unique characteristics of each segment. This research offers an update to the existing foodservice literature on CSR activities.

This study provides some managerial implications to the foodservice industry by focusing on aspects and delivering types of foodservice companies’ CSR activities. In reality, according to National Restaurant Association, top 20 food trends in 2021 are: (1) “new cuts of meat”; (2) “house-made condiments”; (3) “street food-inspired dishes”; (4) “ethnic-inspired breakfast items”; (5) “sustainable seafood”; (6) “healthful kids’ meals”; (7) “vegetable carb substitutes”; (8) “uncommon herbs”; (9) “authentic ethnic cuisine”; (10) “ethnic spices”; (11) “Peruvian cuisine”; (12) “house-made/artisan pickles”; (13) “heritage-breed meats”; (14) “Thai-rolled ice cream”; (15) “African flavors”; (16) “ethnic-inspired kids’ dishes”; (17) “doughnuts with non-traditional filling”; (18) “gourmet items in kids’ meals”; (19) “ethnic condiments”; (20) “ancient grains”. The restaurant industry has already recognized the importance of CSR activities as a trend in 2021. Based on the empirical results and current trends in the foodservice industry, foodservice companies need to emphasize more food ingredients, sourcing, and health than other aspects of CSR activities on their official websites and CSR reports. In particular, from a managerial perspective, foodservice companies need to focus on the COVID-19 situation and consider their customers as victims of Coronavirus, using the high quality of food ingredients, and sourcing and offering high-quality health products as a key solution (or CSR initiatives) for the COVID-19 situation. In addition, foodservice companies can establish and implement food donation events for local communities in need, who have been dramatically affected by the COVID-19 situation, as a long-term CSR initiative. This approach may enable foodservice companies to boost the impacts of CSR activities on customers’ perception, attitudes, and behavior toward their establishments during and even after the era of COVID-19. 

According to the National Restaurant Association, the top 10 concept trends in 2021 were: (1) “hyper-local”; (2) “chef-driven fast casual concepts”; (3) “natural ingredients/clean menus”; (4) “food waste reduction”; (5) “veggie-centric/vegetable-forward cuisine”; (6) “environmental sustainability”; (7) “locally sourced meat and seafood”; (8) “locally sourced produce”; (9) “simplicity/back to basics”; (10) “farm/estate-branded items”. In reality, however, this research found that foodservice companies mostly implemented community-based CSR activities such as charitable donations and corporate giving, by focusing on community welfare. With respect to the CSR perspective, foodservice companies may also consider implementing natural environment and food-focused CSR activities since the top 10 concept trends in 2021 have highlighted the importance of various aspects of CSR activities within the foodservice industry. CSR reports and official websites of the selected foodservice companies provide CSR-related information in a well-detailed and structured manner (e.g., natural environment, food quality, and community; McDonald’s). However, compared to the social media platforms, the CSR reports and official websites may not be popular among young restaurant consumers as a CSR communication channel [36]. More importantly, the process of delivering online information about foodservice companies’ CSR activities to customers may be important to boost the potential influences of the CSR activities on customers’ perceptions, attitudes, and behaviors toward the foodservice companies. From another managerial perspective, this study proposes that social media platforms be used to offer restaurant consumers more access to the CSR reports and official websites. To do so, social media posts should grab restaurant consumers’ attention, leading them to read the CSR reports and to visit the official websites to get more detailed information about restaurant companies’ CSR initiatives. Therefore, foodservice companies need to be more aware of employing online communication tools and using them in an effective manner since compared to traditional communication tools, customers can easily access to any type of information about CSR and tend not to spend much time reading all information about foodservice companies’ CSR activities.

## 6. Conclusions

The main purpose of previous empirical studies in the hospitality sector is to predict positive outcomes from external or internal customers’ perceptions of CSR initiatives [35,37]. However, the conceptual and empirical approaches of the prior CSR research were determined and based on scholars’ perspectives on CSR initiatives instead of foodservice companies’ standpoints on CSR [35,37]. This study attempted to fill the academic gap in CSR hospitality literature by emphasizing what and how foodservice companies are communicating with their various stakeholders. To do so, this study employed a content analysis approach and examined how foodservice companies communicate CSR activities to customers (RQ1), aspects of CSR information foodservice companies provide to their customers (RQ2), types of CSR information foodservice companies provide to their customers (RQ3), and how aspects of CSR activities and types of CSR information differ in two segments (i.e., quick-service vs. dessert cafe) (RQ4). 

Regarding RQ1, compared to other communication tools, official websites with CSR categories were preferred by foodservice companies. With respect to RQ2, several dimensions of community-based CSR activities (i.e., charitable donations, corporate giving, education, and community welfare), natural environment-centered CSR activities (i.e., recycling), marketplace-focused CSR activities (i.e., providing a product of value and relationship with guests), vision and values-related CSR activities (i.e., trust and self-regulation), food-centered CSR activities (i.e., ingredient and materials, food quality and choice, and sourcing), and workforce-oriented CSR activities (i.e., training, compensation and rewards, and diversity and equal opportunity) were identified and measured within the foodservice context. In terms of RQ3, each aspect of foodservice companies’ CSR activities used different types of CSR information (i.e., figures, tables, statistical data, citations, pictures, or text only). Some significant differences between two foodservice segments (i.e., quick-service vs. dessert cafe) were found. More specifically, quick-service restaurants focused more on corporate giving, community welfare, and fairness than dessert cafes. The research questions this study formulated might provide a new standpoint on foodservice companies’ CSR activities and online communication tools management. 

Last, this study proposes some directions for future CSR research in the hospitality industry. This research focused only on the annual CSR reports and official websites of foodservice companies by overlooking the role of their social media pages as a new CSR communication channel. Many foodservice companies have already employed social media platforms as a new CSR communication channel by encouraging their current and prospective consumers to participate in their CSR activities. Thus, future research should investigate CSR-related posts on social media and their impact on hospitality consumers’ responses to CSR initiatives [36]. In addition, future research should consider the potential impact of the ongoing situations on foodservice companies’ CSR initiatives and communication strategies, such as COVID-19 and inflation. Companies in most of the business sectors will attempt to reflect and resolve the ongoing social issues via their CSR initiatives, leading scholars to include the situational factors for future CSR research in the hospitality sector as well.

## Figures and Tables

**Table 1:** Categories of CSR activities (source: Holcomb et al. [1]).

Categories of CSR Activities	Aspects
Community	1. Charitable donations 2. Community welfare 3. Corporate giving 4. Donations in kind 5. Education 6. Grant 7. Local regeneration 8. National welfare 9. Volunteerism 10. World welfare
Environment	1. Cultural heritage 2. Energy management 3. Pollution control 4. Recycle 5. Waste management 6. Water conservation
Marketplace	1. Ethical advertising 2. Providing a product of value 3. Relationship with guests 4. Relationship with suppliers 5. Relationship with shareholders 6. Supplier diversity
Vision and Values	1. Accountability 2. Clear purpose 3. Code of conduct 4. Enduring values 5. Ethical behavior 6. Fairness 7. Self-regulation 8. Trust
Workforce	1. Advancement 2. Fair and equitable benefits 3. Career planning 4. Compensation and rewards 5. Daycare and family accommodations 6. Diversity/equal opportunity 7. Employee assistance program 8. Employee communication 9. Health and safety 10. Recruitment 11. Training
Food	1. Food quality and choice 2. Sourcing 3. Ingredient and materials 4. Food safety

**Table 2:** Research sample (source: *Entrepreneur* Franchise 500 in 2021).

Rank	Company	Website
1	McDonald’s	https://www.mcdonalds.com/us/en-us.html
3	Dunkin’ Donuts	https://www.dunkindonuts.com/en
6	Sonic Drive-In Restaurants	https://www.sonicdrivein.com/
8	Taco Bell	https://www.tacobell.com/
9	Hardee’s	https://www.hardees.com/
11	Jimmy John’s Gourmet Sandwiches	https://www.jimmyjohns.com/
15	Carl’s Jr. Restaurants	https://www.carlsjr.com/
16	Papa John’s Int’l. Inc.	https://www.papajohns.com/
24	Jersey Mike’s Subs	https://www.jerseymikes.com/
25	Marco’s Pizza	https://www.marcos.com/
31	Wingstop Restaurants Inc.	http://www.wingstop.com/
35	Smoothie King	http://www.smoothieking.com/
39	Firehouse Subs	https://www.firehousesubs.com/
41	Baskin-Robbins	https://www.baskinrobbins.com/content/baskinrobbins/en.html
43	Jack in the Box	https://www.jackinthebox.com/
44	Freddy’s Frozen Custard & Steakburgers	https://freddysusa.com/
47	Pizza Hut LLC	https://www.pizzahut.com/
54	Golden Corral Restaurants	https://www.goldencorral.com/
57	Bojangles’ Famous Chicken’n Biscuits	https://www.bojangles.com/
67	Dairy Queen	https://www.dairyqueen.com/us-en/?localechange=1
71	Tropical Smoothie Café	https://www.tropicalsmoothiecafe.com/
76	Denny’s Inc.	https://www.dennys.com/
83	Kona Ice	https://www.kona-ice.com/
94	Kilwins Chocolates Franchise Inc.	http://kilwinsfranchise.com/
95	Blaze Fast-Fire’d Pizza	http://www.blazepizza.com/
98	Cinnabon	https://www.cinnabon.com/
99	Beef Jerky Outlet Franchise Inc.	https://www.beefjerkyoutlet.com/
105	Subway	http://www.subway.com/en-us
112	Arby’s Restaurant Group Inc.	https://arbys.com/
114	McAlister’s Deli	https://www.mcalistersdeli.com/
123	Fuzzy’s Taco Shop	https://www.fuzzystacoshop.com/
124	Auntie Anne’s Hand-Rolled Soft Pretzels	http://www.auntieannes.com/
127	Jet’s Pizza	http://jetspizza.com/
130	HoneyBaked Ham	http://www.honeybaked.com/
140	Checkers and Rally’s Restaurants Inc.	https://www.checkers.com/
153	Chester’s	https://www.chestersinternational.com/
160	Charleys Philly Steak	https://charleys.com/
170	Dickey’s Barbecue Pit	https://www.dickeys.com/
177	Penn Station East Coast Subs	http://www.penn-station.com/
187	Round Table Franchise Corp.	https://roundtablepizzafranchise.com/
188	Del Taco LLc	https://www.deltaco.com/
195	Donatos	https://www.donatos.com/
204	Which Wich Superior Sandwiches	https://www.whichwich.com/
206	Farmer Boys Restaurants	https://www.farmerboys.com/
211	Black Bear Dinners Inc.	https://blackbeardiner.com/
212	Orion Food Systems LLC	http://orionfoods.com/
213	Brass Tap	https://www.brasstapbeerbar.com/
214	Hungry Howie’s Pizza & Subs	https://www.hungryhowies.com/

**Table 3:** Results of intercoder reliability.

CSR Aspects	Cohen’s Kappa
Community	0.812, *p* < 0.001
Environment	0.800, *p* < 0.001
Marketplace	0.792, *p* < 0.001
Vision and values	0.827, *p* < 0.001
Workforce	0.780, *p* < 0.001
Food	0.717, *p* < 0.001
Type of information: community	0.871, *p* < 0.001
Type of information: environment	0.861, *p* < 0.001
Type of information: marketplace	0.837, *p* < 0.001
Type of information: vision and values	0.770, *p* < 0.001
Type of information: workforce	0.879, *p* < 0.001
Type of information: food	0.916, *p* < 0.001

**Table 4:** Communication tool for CSR activities.

Communication Tools	Yes	No
CSR report	14.6% (*n* = 7)	85.4% (*n* = 41)
Independent CSR website	12.5% (*n* = 6)	87.5% (*n* = 42)
Official website with CSR categories	62.5% (*n* = 30)	37.5% (*n* = 18)
Nothing	31.3% (*n* = 15)	68.8% (*n* = 33)

**Table 5:** Aspects of CSR activities: community.

Community	Yes	No
Charitable donations	69.7% (*n* = 23)	30.3% (*n* = 10)
Corporate giving	78.8% (*n* = 26)	21.2% (*n* = 7)
Donations in kind	3.0% (*n* = 1)	97.0% (*n* = 32)
Education	54.5% (*n* = 18)	45.5% (*n* = 15)
Grants	15.2% (*n* = 5)	84.8% (*n* = 28)
Community welfare	78.8% (*n* = 26)	21.2% (*n* = 7)
National welfare	12.1% (*n* = 4)	87.9% (*n* = 29)
World welfare	3.0% (*n* = 1)	97.0% (*n* = 32)
Local regeneration	6.1% (*n* = 2)	93.9% (*n* = 31)
Local volunteerism	39.4% (*n* = 13)	60.6% (*n* = 20)
National level volunteerism	9.1% (*n* = 3)	90.9% (*n* = 30)
World level volunteerism	0.0% (*n* = 0)	100.0% (*n* = 33)

**Table 6:** Aspects of CSR activities: environment.

Environment	Yes	No
Energy management	24.2% (*n* = 8)	75.8% (*n* = 25)
Recycling	27.3% (*n* = 9)	72.7% (*n* = 24)
Water conservation	24.2% (*n* = 8)	75.8% (*n* = 25)
Pollution control	24.2% (*n* = 8)	75.8% (*n* = 25)
Waste management	18.2% (*n* = 6)	81.8% (*n* = 27)

**Table 7:** Aspects of CSR activities: Marketplace.

Marketplace	Yes	No
Relationships with guests	33.3% (*n* = 11)	66.7% (*n* = 22)
Relationships with suppliers	27.3% (*n* = 9)	72.7% (*n* = 24)
Relationships with shareholders	6.1% (*n* = 2)	93.9% (*n* = 31)
Ethical advertising	0.0% (*n* = 0)	100.0% (*n* = 33)
Providing a product of value	72.7% (*n* = 24)	27.3% (*n* = 9)

**Table 8:** Aspects of CSR activities: vision and values.

Vision and Values	Yes	No
Code of conduct	27.3% (*n* = 9)	72.7% (*n* = 24)
Enduring values	24.2% (*n* = 8)	75.8% (*n* = 25)
Self-regulation	36.4% (*n* = 12)	63.6% (*n* = 21)
Ethical behavior	9.1% (*n* = 3)	90.9% (*n* = 30)
Fairness	12.1% (*n* = 4)	87.9% (*n* = 29)
Trust	51.5% (*n* = 17)	48.5% (*n* = 16)

**Table 9:** Aspects of CSR activities: food.

Food	Yes	No
Sourcing	48.5% (*n* = 16)	51.5% (*n* = 17)
Ingredient and materials	60.6% (*n* = 20)	39.4% (*n* = 13)
Food quality and choice	57.6% (*n* = 19)	42.4% (*n* = 14)
Food safety	39.4% (*n* = 13)	60.6% (*n* = 20)

**Table 10:** Aspects of CSR activities: workforce.

Food	Yes	No
Career planning	33.3% (*n* = 11)	66.7% (*n* = 22)
Training	42.4% (*n* = 14)	57.6% (*n* = 19)
Employee assistance program	30.3% (*n* = 10)	69.7% (*n* = 23)
Compensation and rewards	39.4% (*n* = 13)	60.6% (*n* = 20)
Daycare and family accommodations	9.1% (*n* = 3)	90.9% (*n* = 30)
Health and safety	21.2% (*n* = 7)	78.8% (*n* = 21)
Fair and equitable benefits	30.3% (*n* = 10)	69.7% (*n* = 23)
Diversity and equal opportunity	36.4% (*n* = 12)	63.6% (*n* = 21)
Employee communication	33.3% (*n* = 11)	66.7% (*n* = 22)

**Table 11:** Types of information.

Types of Information (Community)	Yes	No
Figures	24.2% (*n* = 8)	75.8% (*n* = 25)
Tables	3.0% (*n* = 1)	97.0% (*n* = 32)
Statistical data	30.3% (*n* = 10)	69.7% (*n* = 23)
Citations	21.2% (*n* = 7)	78.8% (*n* = 26)
Pictures	63.6% (*n* = 21)	36.4% (*n* = 12)
Text only	36.4% (*n* = 12)	63.6% (*n* = 21)
**Types of Information (Environment)**	**Yes**	**No**
Figures	3.0% (*n* = 1)	97.0% (*n* = 32)
Tables	12.1% (*n* = 4)	87.9% (*n* = 29)
Statistical data	27.3% (*n* = 9)	72.7% (*n* = 24)
Citations	0.0% (*n* = 0)	100.0% (*n* = 33)
Pictures	21.2% (*n* = 7)	78.8% (*n* = 27)
Text only	9.1% (*n* = 3)	90.9% (*n* = 30)
**Types of Information (Marketplace)**	**Yes**	**No**
Figures	0.0% (*n* = 0)	100.0% (*n* = 33)
Tables	0.0% (*n* = 0)	100.0% (*n* = 33)
Statistical data	6.1% (*n* = 2)	93.9% (*n* = 31)
Citations	6.1 (*n* = 2)	93.9% (*n* = 31)
Pictures	21.2% (*n* = 7)	78.8% (*n* = 26)
Text only	27.4% (*n* = 9)	72.7% (*n* = 24)
**Types of Information (Vision and Values)**	**Yes**	**No**
Figures	0.0% (*n* = 0)	100.0% (*n* = 33)
Tables	0.0% (*n* = 0)	100.0% (*n* = 33)
Statistical data	0.0% (*n* = 0)	100.0% (*n* = 33)
Citations	6.1% (*n* = 2)	93.9% (*n* = 31)
Pictures	15.2% (*n* = 5)	84.8% (*n* = 28)
Text only	33.3% (*n* = 11)	66.7% (*n* = 22)
**Types of Information (Food)**	**Yes**	**No**
Figures	9.1% (*n* = 3)	90.9% (*n* = 30)
Tables	12.1% (*n* = 4)	87.9% (*n* = 29)
Statistical data	30.3% (*n* = 10)	69.7% (*n* = 23)
Citations	3.0% (*n* = 1)	97.0% (*n* = 32)
Pictures	30.3% (*n* = 10)	69.7% (*n* = 23)
Text only	27.3% (*n* = 9)	72.7% (*n* = 24)
**Types of Information (Workforce)**	**Yes**	**No**
Figures	6.1% (*n* = 2)	93.9% (*n* = 31)
Tables	3.0% (*n* = 1)	97.0% (*n* = 32)
Statistical data	6.1% (*n* = 2)	93.9% (*n* = 31)
Citations	18.2% (*n* = 6)	81.8% (*n* = 27)
Pictures	24.2% (*n* = 8)	75.8% (*n* = 25)
Text only	33.3% (*n* = 11)	66.7% (*n* = 22)

**Table 12:** Results of chi-square analysis.

Segment (Quick-Service vs. Dessert Cafe)	Pearson Chi-Square	Degree of Freedom	*p*-Value
Corporate giving	3.636	1	0.057
Community welfare	3.636	1	0.057
Fairness	3.098	1	0.078
Type of information: Community—statistical data	4.591	1	0.032
Type of information: Community—picture	6.991	1	0.008
Type of information: Environment—table	3.098	1	0.078
Type of information: Food—table	3.098	1	0.078

## Data Availability

Not applicable.
